# Severe hematologic toxicity after low-dose methotrexate in ectopic pregnancy: role of MTHFR polymorphism and drug interaction - a case report and literature review

**DOI:** 10.3389/fphar.2025.1671369

**Published:** 2025-10-02

**Authors:** Xiaojie Huang, Fangjie He, Yanhong Deng, Shengying Shi

**Affiliations:** ^1^ Department of Clinical Pharmacy, Jieyang People’s Hospital, Jieyang, China; ^2^ Department of Pharmacy, The Maternal and Children Healthcare Hospital (Huzhong Hospital) of Huadu, Guangzhou, China; ^3^ Department of Pharmacy, Guangdong Provincial Key Laboratory of Major Obstetric Diseases, Guangdong Provincial Clinical Research Center for Obstetrics and Gynecology, The Third Affiliated Hospital, Guangzhou Medical University, Guangzhou, China

**Keywords:** methotrexate, ectopic pregnancy, MTHFR polymorphism, drug interaction, hematologic toxicity, case report

## Abstract

**Background:**

Low-dose methotrexate (MTX) is a standard treatment for ectopic pregnancy. While generally safe, it can rarely cause life-threatening hematologic toxicity. The mechanisms underlying these severe reactions in patients without traditional risk factors are poorly understood. We report a case of severe pancytopenia and systematically analyze the literature to characterize this rare but critical complication in MTX treatment for ectopic pregnancy.

**Case Presentation:**

A 24-year-old woman received a single 50 mg/m^2^ dose of MTX for a persistent ectopic pregnancy while on concurrent benzathine penicillin therapy for syphilis. Within 24 h, she developed nausea, vomiting, and facial edema, rapidly progressing to severe mucositis and life-threatening pancytopenia with absolute neutrophil count nadir of 0.1 × 10^9^/L, platelet nadir of 8 × 10^9^/L and hemoglobin nadir of 76 g/L. Investigations revealed delayed MTX clearance and a heterozygous methylenetetrahydrofolate reductase (MTHFR) C677T polymorphism, a known genetic risk factor for MTX toxicity. A diagnosis of MTX toxicity was made, and she fully recovered after intensive supportive care including hydration, urine alkalinization, calcium leucovorin rescue, hematopoietic growth factors, and antibiotics.

**Conclusion:**

Severe MTX toxicity following low-dose treatment for ectopic pregnancy is a rare but potentially fatal complication, with 3 deaths among 16 reviewed cases. Our analysis suggests a multifactorial etiology involving genetic predisposition and pharmacokinetic interactions. The MTHFR C677T variant compromises folate metabolism, while concurrent medications like benzathine penicillin may impair MTX renal clearance through competitive inhibition of organic anion transporters. Early symptom onset precedes standard monitoring schedules, necessitating enhanced clinical vigilance and consideration of pharmacogenetic factors and drug interactions in clinical practice.

## 1 Introduction

Ectopic pregnancy (EP) remains a significant cause of maternal morbidity and mortality in the first trimester, with an incidence of 1%–2% among all pregnancies (Panelli et al., 2015). Methotrexate (MTX), a folic acid antagonist, has become a cornerstone of medical management for hemodynamically stable patients with an unruptured ectopic pregnancy (Mullany et al., 2023). Standard treatment protocols include single-dose, two-dose, and multi-dose regimens (Brady, 2017). The most common single-dose regimen involves one intramuscular injection of 50 mg per square of body surface area (50 mg/m^2^), while multi-dose regimens typically involve up to four doses of 1 mg/kg, alternating with leucovorin rescue (Mullany et al., 2023; Brady, 2017). These regimens are classified as “low-dose” MTX therapy, a category that also encompasses the weekly doses of 5–25 mg commonly prescribed in rheumatoid arthritis, where MTX serves as the anchor drug (Katturajan et al., 2021; Romão et al., 2014). Despite long-term and extensive use in this population, severe hematologic toxicity remains rare (Romão et al., 2014), underscoring the overall safety of low-dose MTX. In contrast, high-dose MTX protocols (>500 mg/m^2^), primarily employed in oncology for conditions such as acute lymphoblastic leukemia and osteosarcoma, are well known to carry substantial risks of severe toxicity (Howard et al., 2016). However, the management of high-dose therapy is standardized with prophylactic measures, including hydration, urine alkalinization, therapeutic drug monitoring, and timely leucovorin rescue, which effectively mitigate the occurrence of severe adverse reactions (Howard et al., 2016).

Given this established safety profile of low-dose MTX, severe and life-threatening toxicity following treatment for ectopic pregnancy represents a rare but documented paradox (Isaacs et al., 1996; Kelly et al., 2006; Dasari and Sagili, 2012; Willner et al., 2014; Soysal et al., 2016; Gaïes et al., 2016; Dündar et al., 2017; Shao et al., 2018; Bayraktar, 2021; Sh et al., 2021; Refeno et al., 2021; Yu et al., 2021; Zhang et al., 2023; Stavros et al., 2024). Cases of profound myelosuppression, severe mucositis, and even death have been reported (Kelly et al., 2006; Gaïes et al., 2016; Refeno et al., 2021). The underlying mechanisms for such idiosyncratic and severe reactions remain poorly elucidated, as they often arise in patients who seemingly lack major predisposing risk factors—such as renal dysfunction, third-space fluid accumulation (ascites or pleural effusion), volume depletion, acidic urine, advanced age (>75 years), folate deficiency, hypoalbuminemia, or dosing errors (Jafari et al., 2023).

Growing evidence suggests that this inter-individual variability in drug response may be attributed to a combination of genetic predisposition and external factors (Giletti and Esperon, 2018). Pharmacogenetic studies, primarily in oncology and rheumatology, have identified polymorphisms in genes encoding folate pathway enzymes, most notably methylenetetrahydrofolate reductase (MTHFR), as significant predictors of MTX toxicity (Giletti and Esperon, 2018; Fisher and Cronstein, 2009; Yang et al., 2012). The MTHFR C677T variant results in reduced enzyme activity and has been associated with increased MTX toxicity in these populations. However, the role of these genetic variants in low-dose MTX treatment for ectopic pregnancy remains largely unexplored (Yu et al., 2021). Furthermore, concomitant administration of drugs that interfere with the renal excretion of MTX can lead to delayed clearance and drug accumulation, thereby potentiating its toxic effects (Jafari et al., 2023). MTX is predominantly eliminated through renal mechanisms involving active tubular secretion via organic anion transporters, making it susceptible to drug interactions that compete for these transport pathways (Jafari et al., 2023; Takeda et al., 2002; Hwang et al., 2024).

Here, we report a case of life-threatening pancytopenia in a young woman following a standard single dose of MTX for a persistent ectopic pregnancy. We explore the potential synergistic contribution of an MTHFR polymorphism and a drug-drug interaction with benzathine penicillin. Additionally, we provide a systematic analysis of all reported cases of severe MTX toxicity in ectopic pregnancy treatment to better characterize this rare but critical complication and inform clinical practice regarding risk assessment and management strategies.

## 2 Case Presentation

**FIGURE 1:**
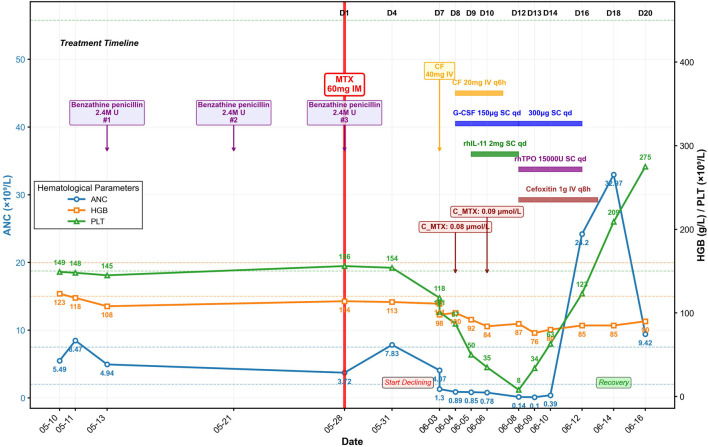
Timeline of key laboratory parameters and therapeutic interventions.

A 24-year-old nulligravid woman (40.4 kg) with no significant prior medical history had undergone laparoscopic salpingotomy for tubal ectopic pregnancy on 10 May 2025 with appropriate initial β-hCG decline (Table 1). She was concurrently receiving intramuscular benzathine penicillin therapy (2.4 million U) for syphilis treatment, with three doses administered on May 13, 21, and 28. However, rising β-hCG levels from 176.8 IU/L on May 21 to 183.8 IU/L on May 28 indicated persistent ectopic pregnancy. Prior to MTX administration, the patient’s baseline liver enzymes, renal function, and serum albumin were all within normal limits, with an aspartate aminotransferase of 17 U/L, alanine aminotransferase of 12 U/L, creatinine of 61 μmol/L, and albumin of 43.1 g/L. Therefore, she received a single intramuscular injection of MTX (50 mg/m^2^, 60 mg) on May 28 (designated as Day 1), administered on the same day as her third benzathine penicillin dose (Table 1; Figure 1).

**TABLE 1:** Serial laboratory parameters of the patient throughout the clinical course.

Date	WBC (10^9^/L)	ANC (10^9^/L)	HGB (g/L)	PLT (10^9^/L)	ALT (IU/L)	Cr (μmol/L)	β-hCG (IU/L)	MTX (μmol/L)
2025/5/10	7.89	5.49	123	149	11	54	8404	
2025/5/11	9.71	8.47	118	148			3317	
2025/5/13	7.53	4.94	108	145			735.5	
2025/5/21							176.8	
2025/5/28 (D1)	6.72	3.72	114	156	12	61	183.8	
2025/5/31 (D4)	9.92	7.83	113	154	11	115	152.5	
2025/6/3 (D7)	5.48	4.07	111	118	22	93		
2025/6/3 (D7)	2.07	1.3	98	101	14	86		
2025/6/4 (D8)	1.64	0.89	100	87	15.7	91	47.12	0.08
2025/6/5 (D9)	2.19	0.85	92	50	13	78		
2025/6/6 (D10)	1.78	0.78	84	35	21.4	75		0.09
2025/6/8 (D12)	1.46	0.14	87	8	15.9			
2025/6/9 (D13)	1.05	0.10	76	34				
2025/6/10 (D14)	2.05	0.39	80	63	9	69		
2025/6/12 (D16)	30.69	24.20	85	123	9.6	66		
2025/6/14 (D18)	39.04	32.97	85	209		61		
2025/6/16 (D20)	14.29	9.42	90	275		54	0.367	

Within 24 h of MTX injection (Day 2), the patient developed nausea, vomiting, pruritus, and hand edema. Her condition rapidly deteriorated with progressive facial edema and severe oral and labial mucositis. On Day 7, as facial edema and pharyngalgia intensified, her blood counts declined precipitously: the white blood cell (WBC) count fell from 5.48 × 10^9^/L to 2.07 × 10^9^/L within hours, with parallel drops in hemoglobin (HGB, 111 to 98 g/L) and platelets (PLT, 118 to 101 × 10^9^/L) (Table 1; Figure 1). After receiving a single 40 mg dose of calcium leucovorin (CF), she was emergently transferred to our institution.

Upon admission on Day 8, the patient was febrile (37.9 °C) and tachycardic. Physical examination revealed severe facial edema, extensive oral ulcerations, and a diffuse maculopapular rash. Laboratory results revealed significant myelosuppression (WBC 1.64 × 10^9^/L; absolute neutrophil count [ANC] 0.89 × 10^9^/L; PLT 87 × 10^9^/L) and impaired renal function (serum creatinine 91 μmol/L). Serum MTX level was 0.08 μmol/L.

The Naranjo Adverse Drug Reaction Probability Scale yielded a score of 8, indicating “probable” MTX-related toxicity. Immediate supportive care was instituted, including intravenous hydration, urine alkalinization, CF rescue (20 mg intravenously every 6 h). Prophylactic granulocyte colony-stimulating factor (G-CSF, 150 µg daily, subcutaneous) was started for neutropenia prevention and recombinant human interleukin-11 (rhIL-11, 2 mg daily, subcutaneous) was initiated to stimulate platelet production and treat thrombocytopenia (Figure 1). Despite these measures, her condition deteriorated. A follow-up serum MTX level on Day 10 was 0.09 μmol/L. On Day 12, she developed neutropenic fever (38.4 °C) with severe myelosuppression (ANC 0.14 × 10^9^/L; PLT nadir 8 × 10^9^/L) and elevated inflammatory markers (C-reactive protein, CRP, 56.82 mg/L; serum amyloid A, SAA, >300 mg/L; procalcitonin, PCT, 0.191 ng/mL). Management was escalated with broad-spectrum antibiotics (cefoxitin), increased G-CSF dosage (300 μg daily), and addition of recombinant human thrombopoietin (rhTPO, 15,000 units daily, subcutaneous) for severe thrombocytopenia, along with platelet transfusions. Her ANC reached its absolute nadir on Day 13 at 0.10 × 10^9^/L.

Following intensified treatment, hematopoietic recovery began. By Day 16, a significant rebound leukocytosis was observed (WBC, 30.69 × 10^9^/L), and platelet counts recovered to 123 × 10^9^/L. Inflammatory markers had also significantly decreased (CRP, 17.63 mg/L; SAA, 76.9 mg/L; PCT, 0.180 ng/mL). Hematopoietic growth factors were discontinued, and antibiotics were completed on Day 17. Genetic testing revealed MTHFR C677T (heterozygous, C/T), MTHFR A1298C (wild-type, A/A), and methionine synthase reductase (MTRR) A66G (heterozygous, A/G) genotypes.

The patient’s clinical condition steadily improved with resolution of mucositis and normalization of renal function. On Day 20, the WBC count was 14.29 × 10^9^/L, the PLT count was 275 × 10^9^/L, and the β-hCG level had fallen to 0.367 IU/L. She was discharged in stable condition on Day 21.

## 3 Discussion

Although low-dose MTX therapy for ectopic pregnancy is generally safe, the severe hematologic toxicity in this case demonstrates that even standard doses can cause life-threatening complications. MTX inhibits dihydrofolate reductase to block DNA synthesis in rapidly dividing trophoblastic cells, but can also affect normal proliferating tissues such as bone marrow and gastrointestinal mucosa (Stika, 2012). This case of life-threatening pancytopenia following standard single-dose MTX therapy highlights the need for heightened clinical awareness of this rare but potentially fatal complication.

### 3.1 Clinical profile of severe MTX toxicity in EP: analysis of the 16-case cohort

To comprehensively characterize this rare but serious complication, we conducted a systematic analysis of 16 cases, including our own, of severe toxicity following low-dose MTX treatment for ectopic pregnancy reported in the literature. A summary of the 16 cases is presented in Table 2, with detailed case characteristics available in Supplementary Table S1, and statistical analysis results are shown in Table 3.

**TABLE 2:** Summary of severe MTX toxicity cases following low-dose treatment for EP.

Case	Year	Age	MTX dosing regimen	Initial symptoms (onset)	Nadir blood counts^a^	MTX level (µmol/L)	MTHFR polymorphism	Suspected drug interactions	Outcome
1 (Isaacs et al., 1996)	1996	23	Single dose, 50 mg/m^2^, IM	Mucositis, pruritic rash, nausea, vomiting, fever (D3)	ANC: 0.3 (D11) PLT: 17 (D11) HGB: 67 (D11)	—	—	—	Recovered
2 (Isaacs et al., 1996)	1996	32	Three doses (D1, 3, 5), 1 mg/kg, IM	Severe mucositis, fever (D6)	ANC: 0.13 (D8)	—	—	—	Recovered
3 (Kelly et al., 2006)	2006	Young	Single dose, 50 mg/m^2^, IV	Abdominal discomfort, nausea, vomiting, severe oral pain (D5)	WBC: 0.4 (D9) PLT: 10 (D20) HGB: 77 (D5)	0.11 (D3) 0.10 (D5) 0.08 (D7) 0.07 (D9) 0.02 (D11)	—	—	Died
4 (Dasari and Sagili, 2012)	2012	25	Four doses (D1, 3, 5, 7), 50 mg, IM	Fever, vomiting (D7); oral ulcers, melena (D8)	ANC: <0.1 (D12) PLT: 13 (D14) HGB: 68 (D13)	—	—	—	Recovered
5 (Willner et al., 2014)	2014	21	Single dose, 100 mg, IV	Fever, sore throat, pruritus, maculopapular rash, mucositis (D2)	WBC: 0.73 (D13) ANC: 0.02 (D13) PLT: 9.9 (D15) HGB: 79.9 (D10)	0.12 (D4) 0.13 (D5) 0.07 (D8) 0.02 (D10)	—	—	Recovered
6 (Soysal et al., 2016)	2016	38	Two doses (D1,7), 50 mg/m^2^, IM	Bloody diarrhea, oral lesions, macular rash (D12)	WBC: 0.3 (D16) PLT: 37 (D16) HGB: 62 (D12)	0.0044 (D12)	—	—	Recovered
7 (Gaï1es et al., 2016)	2016	32	Single dose, 44 mg/m^2^, IM	Mucositis, odynophagia (D3); fever, diffuse erythema (D9)	WBC: 0.3 (D9) PLT: 14 (D9) HGB: 77 (D9)	0.05 (D11) 0.03 (D13)	—	—	Died
8 (Dündar et al., 2017)	2017	33	Single dose, 50 mg/m^2^, IM	Severe nausea, vomiting (D2)	WBC: 1.2 (D10) ANC: 0.3 (D10) PLT: 50 (D13) HGB: 81 (D14)	—	—	—	Recovered
9 (Shao et al., 2018)	2018	27	Single dose, 50 mg, IM	Severe vomiting, mucositis (D4); fever (D6)	WBC: 0.7 (D6) PLT: 11 (D12) HGB: 80 (D10)	<0.02 (D7)	—	—	Recovered
10 (Bayraktar, 2021)	2021	29	Single dose, 50 mg/m^2^, IM	Generalized erythema, mucositis, fever (D4)	WBC: 4.1 (D4) ANC: 0.7 (D4) PLT: 174 (D4) HGB: 105 (D4)	—	—	—	Recovered
11 (Shafie et al., 2021)	2021	30	Single dose, 50 mg/m^2^, IM	Pustular rash, alopecia, mucositis, AKI, fever (D2)	WBC: 0.1 (D6) PLT: 10 (D6) HGB: 66 (D10)	0.36 (D1) 0.07 (D4) 0.03 (D9)	—	—	Recovered
12 (Refeno et al., 2021)	2021	30	Single dose, 1 mg/kg, IM	Diffuse rash, fever, nausea, fatigue, jaundice, diarrhea (D3)	ANC: 0.1 (D14) PLT: 32 (D14) HGB: 108 (D14)	—	—	—	Died
13 (Yu et al., 2021)	2021	38	Single dose, 75 mg, IM	Nausea, vomiting, diarrhea, fever, sore throat (D2)	WBC: 0.22 (D10) ANC: 0 (D11) PLT: 4 (D16) HGB: 65 (D20)	0.04 (D7) 0.013 (D11) 0.010 (D13)	MTHFR TT (C677T) MTHFR AA (A1298C)	—	Recovered
14 (Zhang et al., 2023)	2023	46	Single dose, 50 mg, IM	Nausea, vomiting, diarrhea (D2)	WBC: 2.53 (D16) ANC: 1.00 (D17) PLT: 125 (D8) HGB: 95 (D5)	0.262 (D7) 0.116 (D11) 0.06 (D14)	MTHFR TT (C677T) ABCB1 (T3435C)	—	Recovered
15 (Stavros et al., 2024)	2024	23	Single dose, 50 mg/m^2^, IM (Intragestational Injection)	Oral ulcers, fever, pruritic maculopapular rash (D4)	WBC: 0.3 (D7) ANC: 0.1 (D6) PLT: 30 (D7) HGB: 68 (D7)	—	—	—	Recovered
16	2025	24	Single dose, 50 mg/m^2^, IM	Nausea, vomiting, pruritus, facial edema, oral ulcers (D2)	WBC: 1.05 (D13) ANC: 0.1 (D13) PLT: 8 (D12) HGB: 76 (D13)	0.08 (D8) 0.09 (D10)	MTHFR CT (C677T) MTHFR AA (A1298C) MTRR AG (A66G)	Benzathine penicillin	Recovered

^a^ Values are expressed as 10^9^/L for WBC, ANC, and PLT, and g/L for HGB.

**TABLE 3:** Statistical analysis of clinical features and outcomes in severe MTX toxicity cases.

Characteristic	Data available (n)	Value
Age (years), Mean ± SD	15	30.1 ± 6.8
Reproductive History, n (%)	12	
Nulligravida		5 (41.7)
Parous		7 (58.3)
Comorbidities, n (%)	16	
Chronic kidney disease/hemodialysis		2 (12.5)
History of ectopic pregnancy		3 (18.8)
Previous pelvic surgery		3 (18.8)
Allergy history		3 (18.8)
Ectopic Pregnancy Characteristics	15	
Initial β-hCG (IU/L)^a^, Median (IQR)		1670 (189–2851)
MTX Dosing Regimen, n (%)	16	
Single-dose		13 (81.3)
Multiple-dose		3 (18.8)
Toxicity Manifestations		
Time to onset of initial symptoms (days), median (IQR)	16	2 (1–3)
Hematologic Toxicity^b^ (CTCAE 5.0)		
Neutropenia, n (%)	11	
Grade 1 (LLN ^c^–1.5 × 10^9^/L)		0 (0.0)
Grade 2 (<1.5–1.0 × 10^9^/L)		1 (9.1)
Grade 3 (<1.0–0.5 × 10^9^/L)		1 (9.1)
Grade 4 (<0.5 × 10^9^/L)		9 (81.8)
Thrombocytopenia^d^, n (%)	15	
Grade 1 (LLN–75 × 10^9^/L)		0 (0.0)
Grade 2 (<75–50 × 10^9^/L)		1 (6.7)
Grade 3 (<50–25 × 10^9^/L)		3 (20.0)
Grade 4 (<25 × 10^9^/L)		9 (60.0)
Anemia, n (%)	15	
Grade 1 (LLN–100 g/L)		2 (13.3)
Grade 2 (<100–80 g/L)		3 (20.0)
Grade 3 (<80 g/L)		10 (66.7)
Grade 4 (Life-threatening consequences)		0 (0.0)
Other Toxicities, n (%)	16	
Gastrointestinal Toxicity		16 (100.0)
Dermatologic toxicity		13 (81.3)
Hepatotoxicity		4 (25.0)
Renal toxicity		6 (37.5)
Laboratory Parameters at Nadir		
Time to nadir (days)^e^, Median (IQR)	16	10 (7–12)
Time to hematologic recovery (days)^f^, Median (IQR)	14	14 (12–16)
Neutrophil count at nadir (×10^9^/L), Median (IQR)	11	0.10 (0.10–0.30)
Platelet count at nadir (×10^9^/L), Median (IQR)	15	14 (10–37)
Hemoglobin at nadir (g/L), Median (IQR)	15	77 (67–81)
Treatment and Supportive Care		
Leucovorin rescue therapy, n (%)		12 (75.0)
G-CSF administration, n (%)		13 (81.3)
Blood product transfusion, n (%)		10 (62.5)
Antibiotic therapy, n (%)		15 (93.8)
Renal replacement therapy^g^, n (%)		4 (25.0)
Genetic and Drug Monitoring		
MTX serum concentration measured, n		9
MTHFR gene testing performed, n		3
MTHFR C677T TT genotype, n		2
MTHFR C677T CT genotype, n		1
MTHFR A1298C AA genotype, n		2
Clinical Outcomes		
Complete recovery, n (%)		13 (81.3)
Deaths, n (%)		3 (18.8)

^a^ Measured before MTX treatment.

^b^ Toxicity grading is based on CTCAE v5.0.

^c^ LLN, lower limit of normal.

^d^ Two patients with platelet counts >100 × 10^9^/L were considered normal.

^e^ Time to lowest leukocyte/neutrophil count.

^f^ For patients with grade 4 neutropenia (ANC <0.5 × 10^9^/L), defined as time to ANC recovery >0.5 × 10^9^/L; for patients without severe neutropenia, defined as time to initial rise in WBC or ANC from nadir.

^g^ Two patients were on maintenance dialysis pre-treatment.

The cohort had a mean age of 30.1 ± 6.8 years, with median initial β-hCG levels of 1670 IU/L (Interquartile Range, IQR, 189–2851 IU/L), well within the range where medical management with MTX is considered effective (Hendriks et al., 2020). The vast majority of patients (13 of 16, 81.3%) received the standard single-dose MTX regimen, reinforcing that toxicity was not due to unusual dosing schedules. Of critical importance, only two patients (Case 3 (Kelly et al., 2006) and Case 5 (Willner et al., 2014)) had pre-existing chronic kidney disease requiring maintenance hemodialysis, providing a clear pharmacokinetic basis for their adverse outcomes given MTX’s predominant renal elimination. However, for the majority of the cohort without documented renal disease, other explanatory factors must be sought.

The clinical presentation was characterized by profound severity and multi-system involvement. Universal gastrointestinal toxicity occurred in all patients, typically manifesting as severe mucositis, stomatitis, nausea and vomiting. Dermatologic toxicity developed in 81.3% of patients, presenting as rashes or skin ulcerations. This universal and severe impact on mucosal surfaces and rapidly dividing cells is a hallmark of systemic MTX poisoning. The most striking feature was extreme myelosuppression: grade 4 neutropenia (ANC <0.5 × 10^9^/L) occurred in 81.8% of patients with available data, grade 4 thrombocytopenia (PLT <25 × 10^9^/L) in 60.0%, and severe anemia (grade 3, HGB <80 g/L) in 66.7%. The median nadir values were life-threatening: neutrophils 0.10 × 10^9^/L (IQR, 0.10–0.30) and platelets 14 × 10^9^/L (IQR, 10–37). This degree of pancytopenia places patients at extreme risk for overwhelming sepsis and spontaneous hemorrhage, more aligned with outcomes after myeloablative chemotherapy than a single 50 mg/m^2^ MTX dose (Howard et al., 2016).

The temporal progression highlights a critical patient safety issue. Initial symptoms onset occurred rapidly with a median of just 2 days (IQR, 1–3) following MTX administration, significantly earlier than standard follow-up schedules. ACOG-endorsed protocols typically involve β-hCG measurements on days 4 and 7 post-injection (Obstetricians, 2018), creating a potential 2–3 days window during which patients could develop life-threatening toxicity at home while attributing early symptoms to expected medication effects.

The clinical course was severe and protracted, with the hematologic nadir occurring at a median of 10 days (IQR, 7–12) after MTX administration, and recovery requiring 14 days (IQR, 12–16) from the time of MTX administration for survivors. Despite aggressive supportive care measures, the mortality rate was 18.8% (3 deaths), underscoring the overwhelming nature of the toxic cascade once initiated in susceptible individuals.

### 3.2 MTHFR gene polymorphisms and MTX toxicity

Within the MTX metabolic pathway, polymorphisms in the MTHFR gene are the most extensively studied genetic markers associated with MTX toxicity (Fisher and Cronstein, 2009; Song et al., 2021). MTHFR is a critical enzyme in folate metabolism, catalyzing the irreversible conversion of 5,10-methylenetetrahydrofolate to 5-methyltetrahydrofolate, which is the primary circulatory form of folate and the essential methyl donor for the remethylation of homocysteine to methionine (Zhao et al., 2024). The MTHFR C677T (rs1801133) polymorphism is the genetic variant most robustly associated with MTX toxicity (Giletti and Esperon, 2018). Compared to the wild-type (CC) genotype, heterozygous (CT) individuals have approximately 65% of normal enzyme activity, while homozygous (TT) individuals have their activity drastically reduced to about 30% (Giletti and Esperon, 2018). Multiple studies have consistently demonstrated that the C677T polymorphism is associated with increased overall MTX toxicity (Fisher and Cronstein, 2009; Song et al., 2021; Shao et al., 2017). In contrast to C677T, the MTHFR A1298C (rs1801131) polymorphism has a lesser impact on MTHFR enzyme activity, and its association with MTX toxicity is more controversial in the literature (Giletti and Esperon, 2018). Interestingly, emerging evidence from hematologic malignancies suggests that A1298C may actually confer a protective effect against MTX-induced toxicity, rather than increasing risk (Campbell et al., 2016). This protective pattern contrasts markedly with findings in rheumatoid arthritis populations, where the association between A1298C and MTX toxicity remains inconsistent or non-significant (Giletti and Esperon, 2018). The divergent effects observed across different patient populations suggest that the clinical significance of A1298C polymorphism may be disease-specific and influenced by distinct treatment protocols used in oncology versus rheumatology settings.

However, it is crucial to emphasize that the vast majority of research on MTX pharmacogenomics has been conducted in hematological malignancies and rheumatoid arthritis patients, with virtually no data available specifically for ectopic pregnancy treatment. This represents a significant knowledge gap in the field, largely attributed to the rarity of severe MTX toxicity cases in this clinical setting.

Of the 16 cases of severe toxicity analyzed in the literature, genetic polymorphism data was available for only three cases, including our own. This limited dataset provides valuable preliminary insights into the genetic predisposition patterns in ectopic pregnancy-related MTX toxicity. Case 13 (Yu et al., 2021) presented with the highest-risk MTHFR genetic profile, being homozygous for MTHFR 677 TT and carrying the wild-type MTHFR 1298 AA genotype. Case 14 (Zhang et al., 2023) exhibited the MTHFR 677 TT genotype and carried an additional ABCB1 3435T>C polymorphism affecting drug transport mechanisms. Our case demonstrated a compound pattern with MTHFR 677 CT, MTHFR 1298 AA, and an additional MTRR 66 AG polymorphism affecting the broader folate metabolic pathway.

The consistent finding across all three cases is the presence of at least one high-risk MTHFR C677T ‘T' allele, reinforcing the established association between this polymorphism and MTX toxicity. Notably, cases 13 and 14, both carrying the homozygous 677 TT genotype (associated with only 30% of normal enzyme activity), experienced severe toxicity despite receiving standard low-dose MTX regimens. Our case, with the heterozygous 677 CT genotype (approximately 65% enzyme activity), also developed life-threatening complications.

An intriguing observation from this limited cohort is that two of the three cases (cases 13 and our case) carried the wild-type MTHFR 1298 AA genotype, while the A1298C status of case 14 was not reported. This finding raises important questions about the role of A1298C polymorphism in ectopic pregnancy-related MTX toxicity. Given that previous research in hematologic malignancies has suggested a protective effect of the A1298C variant, the predominance of wild-type AA genotype in our severe toxicity cases may support the hypothesis that the absence of this potentially protective variant could contribute to increased susceptibility. However, given the extremely small sample size and the disease-specific variability of A1298C effects previously observed, definitive conclusions cannot be drawn.

An additional finding of interest in our case was a heterozygous A66G (rs1801394) mutation in the MTRR gene. MTRR plays a vital supportive role in the folate cycle by maintaining the activity of methionine synthase (MTR) through reductive methylation. Therefore, a polymorphism in MTRR could also disrupt the folate pathway. However, research on the MTRR A66G polymorphism’s relationship with MTX toxicity is limited and has yielded inconsistent results (Xu et al., 2022).

### 3.3 Potential drug interactions: Benzathine penicillin and MTX

While genetic polymorphisms represent a significant risk factor for MTX toxicity, potential drug interactions must also be considered as contributing factors. MTX is predominantly eliminated by the kidneys (80%–90%) through glomerular filtration and active tubular secretion via organic anion transporters (hOAT1 and hOAT3) and efflux transporters (BCRP/ABCG2 and MRP2/ABCC2) (Jafari et al., 2023; Wang et al., 2018). Any interference with these transport mechanisms can significantly impair MTX clearance and lead to accumulation and toxicity.

Several drug classes are known to interact with MTX through inhibition of renal transporters, including NSAIDs, proton pump inhibitors, and penicillins, among others (Jafari et al., 2023; Hwang et al., 2024; Wang et al., 2020). These interactions are well-established in high-dose MTX settings (Howard et al., 2016; Levêque et al., 2011), though their clinical significance in low-dose MTX therapy remains controversial (Hall et al., 2017; Svanström et al., 2018).

The interaction between penicillins and MTX has been documented through case reports and pharmacokinetic studies (Hall et al., 2017). Penicillins compete with MTX for active renal tubular secretion by inhibiting OAT1 and OAT3 transporters (Jafari et al., 2023). While high-dose MTX interactions with penicillin derivatives are well-documented (Ronchera et al., 1993; Yamamoto et al., 1997; Zarychanski et al., 2006; Titier et al., 2002; Krämer et al., 2021), evidence for low-dose MTX remains conflicting. A pharmacokinetic study by Herrick et al. found that flucloxacillin did not significantly alter low-dose MTX parameters or cause clinical toxicities in 10 rheumatoid arthritis patients (HERRICK et al., 1996). Besides, case series have described severe adverse outcomes in patients receiving both therapies, but these cases were often confounded by other risk factors including advanced age, renal dysfunction, and hypoalbuminemia (Mayall et al., 1991; Lim et al., 2005).

Our case presents a unique interaction scenario. The patient received benzathine penicillin for syphilis treatment on May 13, 21, and 28, with MTX administered on May 28. Benzathine penicillin is a long-acting depot formulation with a prolonged half-life of 189 h, maintaining therapeutic levels for approximately 23 days (Li et al., 2024). The concurrent administration ensured substantial penicillin concentrations during the critical MTX elimination period.

As a weak organic acid, benzathine penicillin competes with MTX for the same organic anion transporters (OAT1 and OAT3) responsible for active tubular secretion (Jafari et al., 2023). This competitive inhibition can impair MTX clearance and elevate serum concentrations. Clinical evidence supporting this potential interaction was observed in our patient’s delayed MTX clearance, with serum concentrations of 0.08 μmol/L on Day 8 and 0.09 μmol/L on Day 10. For context, while standardized monitoring thresholds for low-dose MTX are not established, high-dose MTX protocols consider concentrations >0.1 μmol/L at 72 h as toxicity-predictive, with monitoring continued until levels fall below 0.05–0.1 μmol/L (Howard et al., 2016; Yu et al., 2021). Our patient’s persistent levels at Days 8–10, though not extremely high compared to high-dose MTX toxicity thresholds, may have been sufficient to trigger severe reactions in an individual with genetic predisposition to compromised folate metabolism due to MTHFR polymorphisms.

## 4 Conclusion

This case demonstrates that severe hematologic toxicity following low-dose methotrexate for ectopic pregnancy, although rare, can be life-threatening even in patients without traditional risk factors. Our analysis of 16 cases reveals an 18.8% mortality rate, with most patients lacking established risk factors except for two on maintenance hemodialysis. The severe toxicity in our case likely resulted from synergistic effects of genetic predisposition (MTHFR C677T polymorphism) and pharmacokinetic interaction with concurrent benzathine penicillin therapy, which reduced folate metabolism capacity and impaired MTX renal clearance through competitive inhibition of organic anion transporters. Critically, toxicity onset occurs rapidly (median 2 days), preceding standard monitoring schedules and creating a dangerous safety gap. Healthcare providers should maintain heightened vigilance for early toxicity signs, particularly in patients with potential genetic predisposition or concurrent medications affecting MTX clearance. Future research should focus on validating genetic risk factors and developing risk stratification strategies to optimize MTX safety in ectopic pregnancy management.

## Data Availability

The original contributions presented in the study are included in the article/Supplementary Material, further inquiries can be directed to the corresponding author.
